# Teaching a Person with Memory Impairment Smartphone Use for Emergencies during Outdoors Walking: Case Report

**DOI:** 10.3390/geriatrics3010008

**Published:** 2018-02-20

**Authors:** Jennifer H. Maze, Linda A. Hunt

**Affiliations:** School of Occupational Therapy, College of Health Professions, Pacific University, Hillsboro, OR 97123, USA; Jen.Maze@pacificu.edu

**Keywords:** community mobility, dementia, occupational therapy, outdoors walking, technology devices

## Abstract

Safety issues arise during the performance of activities as dementia advances. Occupational therapists collaborate with dementia clients and their caregivers to find solutions and strategies to prolong safe activity participation. Additionally, occupational therapists teach through demonstration showing engagement in specific activities may no longer be safe. We present the case of a 70-year-old male with mild dementia. His caregiver believes he needs to use a smartphone for safety during outdoors walking; the client’s enjoyed and valued occupation. This case report illustrates smartphone use may be difficult to learn for a person with mild dementia. It highlights the need for the caregiver and person with dementia to receive education together for best understanding. New technologies for community mobility such as outdoors walking are considered.

## 1. Introduction

Healthcare professionals and caregivers may desire prolonging engagement in activities as long as possible for a person with dementia. Conversely, they may prematurely disengage a person for fear of danger to the person. This case report describes how an occupational therapist worked with a client having dementia and his caregiver by providing intervention to test continued independent outdoors walking by the person with dementia. The intervention strategy was to teach smartphone use. 

It is crucial to provide background information first on people with dementia becoming lost in the community while walking alone for recreation or transportation. We define occupational therapy as a profession that assesses people’s ability to engage in meaningful activities. Safety while performing activities takes precedence. We end with comments about technology and how smartphone use may not be an answer to independence in community mobility for people with dementia.

### 1.1. Dementia and the Role of Occupational Therapy 

Of the estimated 5.5 million Americans living with Alzheimer’s dementia in 2017, an estimated 5.3 million are age 65 and older and approximately 200,000 individuals are under age 65 and have younger-onset Alzheimer’s [1]. Alzheimer’s is a type of dementia that causes problems with memory, thinking and behavior. Symptoms usually develop slowly and get worse over time, becoming severe enough to interfere with daily tasks. The most common early symptom of Alzheimer’s is difficulty remembering newly learned information [2]. This symptom is illustrated through this case report and affects outcomes.

Occupational therapy practitioners work not only with people with dementia, but also their caregivers and family members. Occupational therapy practitioners focus on the person’s remaining abilities. They use adaptations and modifications to maintain client activity participation for as long as possible, making them valuable members of the care team. Primary concern when teaching activity engagement is how safe the person is performing the desired activity [3]. Occupational therapists seek to explore what the client wants to do and how that activity may be modified or enhanced with technology for participation. 

Occupational therapy practitioners use activity analysis in the assessment and intervention process. Activity analysis is defined as examining the typical demands of an activity, the range of skills involved in its performance, and how technology and the environment might be ascribed to enable performance. Occupational therapy practitioners have a unique and holistic perspective to activity analysis as a fundamental component of their practice. The occupational therapy perspective not only looks at how an activity might be typically done but how it is done and experienced by an individual [4,5]. Key to using this approach is that a change in one feature of an activity may change the extent of the demand in another feature. For example, an increase in the number or sequence of steps in an activity increases the demand on attention and memory skills. This last factor was essential to this case report: Can a client with mild dementia remember the steps for using a smartphone even when the process is simplified for use? 

### 1.2. Intentional Walking as Community Mobility 

Community mobility is defined as “planning and moving around in the community and using public or private transportation, such as driving, walking, bicycling, or accessing and riding in buses, taxi cabs, or other transportation systems” [4] (p. S19). Outdoors walking as community mobility is the most common form of physical activity for older adults [5]. Walking requires minimal equipment, and the individual dictates intensity [6]. Outdoors walking may support health by offering opportunities for physical, social, and leisure activities [7]. 

Unfortunately, community mobility may be dangerous for those with dementia. They may become disoriented in unfamiliar environments and later have difficulty finding their way in familiar environments [8]. Functionally, this finding means that drivers and walkers may start out from their home and forget where they intended to go, do not recognize or attend to their own familiar neighborhood streets and landmarks, and consequently become lost. It is known that when driving, becoming lost may result in injury or death [9]. Examples include: “A driver who was lost on a freeway performed a U-turn to turn around, colliding head-on with another driver; a driver who was lost at night accidentally drove the vehicle onto a boat ramp into a lake, and the body was found months later; and a driver who ran out of gas because he had driven for hours trying to find his way home got out of the car to walk and died from exposure to the weather” [10] (p. 226). 

Studies indicate that all persons with dementia are at risk of becoming lost in the community whether driving or walking [8,9,10,11,12,13]. Rowe has been instrumental in pointing out the issues surrounding people with dementia becoming lost near their homes and has published numerous studies that document the dangers of independent community mobility for those having dementia [8,9,10,11,12]. For example, Rowe documents a person with dementia who went on a bike ride, became lost, and was found in a wooded area by a search team about 0.25 miles from his home. Missing for several days, he experienced exposure to the elements and severe dehydration [13]. 

## 2. Case Study: Person with Memory Impairment Desires Continued Engagement in Outdoors Walking

### 2.1. Assessment and Intervention

Carl, a 70-year-old retired male was diagnosed with mild dementia. He reports having a limited external support system, as his closest family members are deceased. He lives in an adult care home and receives in-home occupational therapy services as well as outpatient occupational therapy services through a community-based day center. Carl’s in-home caregiver, Julia provides him with assistance in some activities such as laundry and meal preparation. He uses a ride service to attend outpatient therapy sessions. Carl no longer drives. However, he often walks in his neighborhood and enjoys frequenting the neighborhood park. Walking is his mode of transportation to this neighborhood park. Carl’s daily routine consists of self-care activities, reading, watching television, outdoor walks in his neighborhood, caring for his living quarters and attending a community day center for therapy services, which include occupational therapy and social engagement activities. He enjoys his relationships with the other day program participants as well as his relationship with his rehabilitation therapists.

Julia requested Carl have a safety evaluation. She was particularly concerned Carl would become lost while walking to/from the neighborhood park. Although Julia reported no incidents of Carl wandering, she expressed her concern that he could possibly get lost while walking in the community. She frequently discouraged him from taking walks. Julia reported Carl frequently becomes confused using the television controls in his room. Carl reported frustration with his ability to keep track of his thoughts, stating “I can’t seem to get things done like I used to, but I have a lot of great ideas.” The occupational therapist administered the Trail Making Test [14]. This assessment provided information about visual search speed, scanning, processing speed, mental flexibility, sequencing, and executive functioning. Carl took over 2 min to complete Part 1. He became frustrated while attempting to complete Part 2, putting down the pencil and saying he did not want to continue. Oftentimes, when healthcare providers see clients in the community, they do not have access to hospital records or medical reports. Unfortunately, the clinician did not have all the medical background information regarding this client to report in this paper. We believe that this case report reflects practice in homes and the community as healthcare providers often work without all the background information. They rely on caregiver report for updated information.

The occupational therapist established goals with Carl and Julia to address his personal safety while walking in the community by introducing the use of a smartphone. Both believed it was worth trying despite Julia reporting Carl frequently becomes confused using the television controls in his room. In-home visits focused on teaching Carl to use his smartphone, one that is designed for older adults. These visits also provided Julia with verbal feedback and demonstration regarding his ability to use the smartphone. The therapist matched cognitive level to activity analysis of using a smartphone. The occupational therapist provided simple instructions to use a smartphone when walking outdoors. The simplest was to press the red button if he was lost or in danger. Julia’s phone number was programmed into the phone. It was hoped that Carl would be able to call Julia if he became lost on a walk.

### 2.2. Outcomes

One of the greatest challenges Carl faced was the ability to error correct if he reached an unintended action on the phone. On multiple occasions, Carl expressed frustration with the small size of the phone keys. He was highly distractible during teaching sessions. Furthermore, the smartphone had features that confused Carl such as a text messaging button, camera, photo album, email, and brain games. Carl could not remember the purpose of emergency button.

Julia observed the therapist working with Carl. The last home visit aimed to assess Carl’s use of his smartphone in his home environment as well as address any further caregiver concerns. During multiple attempts at making a call, Carl needed moderate cues from the therapist and completed a call independently on one occasion. The therapist informed Julia that after assessing Carl’s progress, his goals clearly needed to be modified in order to address the safety concerns in a different way. Julia and the therapist discussed the possibility of using a personal tracking device to prevent Carl from getting lost in the community. Julia expressed that she was grateful that they explored smartphone use as she wondered if it would be helpful and did not know how to teach Carl its use. She was not surprised that smartphone use would be challenging for him. Julia and the therapist discussed outstanding considerations such as whether or not Carl would remember to charge his phone and take the phone with him when in the community. Julia believed he might misplace the phone. Handling calls that were the wrong number or soliciting calls received may be challenging. Having the demonstration further resolved the importance of having an identification card and Julia’s phone number on it with Carl when he was walking in the community. She believed with the cold rainy weather coming Carl would stop going for community walks. Hopefully, she could find a companion to go with him or he would no longer desire this activity. They ended the session with Julia agreeing with the therapist’s advice: Julia would obtain a tracker device for Carl. This would alert her if he walks too far and identifies his location, which she stated would bring some peace of mind. 

## 3. Discussion

Enabling independence may improve the quality of life for a person with dementia, but this can only be done when it is generally safe to do so [13,15,16,17]. This case study reveals the challenge for people with dementia to learn and retain novel information regarding technology use that enables desired independence. Its outcomes suggest that viewing the person with dementia and their caregiver as one unit may bring better understanding of what activities should be pursued and which activities require modification or cessation. Further, this case reveals that caregivers of people with dementia may not receive adequate safety information specific to those who are in their care. Hands-on demonstration may be a highly effective process for teaching safety outcomes to caregivers. Demonstration yields best understanding. 

### Technology Use and Dementia

Prior to navigation technology and smartphones, some healthcare workers may have recommended the use of a co-walker as a viable solution to prolong outdoor walking for a person with dementia. Many walkers may enjoy the socialization of this strategy. However, some individuals may find having a walking partner an intrusion of private reflective time. Some people prefer the solitude for quiet time to enjoy nature.

Thinking about this problem in a bigger perspective, research stresses the importance of intelligent design of community environments for individuals at risk for cognitive decline [16]. Dementia friendly community design highlights familiarity, legibility, distinctiveness, accessibility, comfort, and safety. Mitchell’s work shows small street blocks with direct, connected routes and good visual access, varied urban form, and architectural features, and distinctive, unambiguous environmental cues could enhance successful orientation of one’s position, and planning and following a route [17].

However, this land-use planning for dementia does not solve immediate needs for people with dementia and their caregivers’ concerns about safety. This brings reliance on technology. Although teaching the use of a smartphone may not be workable, other technology may provide some safety options. Rowe et al. describes a person who was lost 0.25 miles from his home for 6 days. The search for him required law enforcement, the use of a helicopter search, and the work of volunteers [13]. Wearing a locator device may have helped identify the lost person’s location immediately. This device could have saved resources, finding this lost man quickly to avoid injury and possible death. Alzheimers.net provides an overview of such devices [18]. Healthcare providers need to be knowledgeable about technology and educate caregivers on the necessity of using such technology. Unfortunately, this case report is limited as only one smartphone was available for teaching purposes with Carl. Technology may require trial and error to discover which device would work best for a given timeframe. What works today may not work tomorrow. People teaching technology to a person with dementia must also explain to the client and caregiver that the technology use will change with the progression of the disease and that re-evaluation is necessary for safety concerns.

## 4. Conclusions

Sometimes, the quest for independence versus safety drives healthcare professionals or caregivers to promote or discourage engagement in an activity without knowing if partaking is safe or not. This case report illustrates the safety risks of what may be taught for independent participation and what may become a safety hazard for those with memory impairments. Occupational therapists are in a unique position to demonstrate to caregivers and clients with dementia activities that may be dangerous when there is memory impairment. Demonstrating to both at once may yield best understanding. Having a variety of technology samples to teach may demonstrate best what choices need to be made for safety in the community and if this option is possible. The occupational therapy intervention did not change the client’s behavior as others have found [19]. However, the occupational therapist was able to provide the caregiver with a better understanding of the client’s current abilities.
